# A Rare Cause of Cardiomyopathy: A Case of Selenium Deficiency Causing Severe Cardiomyopathy that Improved on Supplementation

**DOI:** 10.7759/cureus.1627

**Published:** 2017-08-29

**Authors:** Cyrus M Munguti, Mahmoud Al Rifai, Wassim Shaheen

**Affiliations:** 1 Internal Medicine, University of Kansas School of Medicine - Wichita; 2 Heartland Cardiology, University of Kansas School of Medicine - Wichita

**Keywords:** selenium induced cardiomyopathy, nutritional cardiomyopathy, heart failure, selenium

## Abstract

Selenium-associated cardiomyopathy is a rare but potentially fatal condition that has previously been described in the literature. Once identified, the condition may be reversible via supplementation. Gastrointestinal operations, especially bariatric and bowel resection, are increasingly associated with multiple deficiencies, including a deficiency of the micronutrient selenium (Se). This case report presents a patient with Se-deficient cardiomyopathy whose condition improved due to Se replacement.

## Introduction

Selenium (Se) is a micronutrient present in various foods, including seafood, meats, fish, and grains. The daily requirement in humans is about 55 µg/day, and most Western diets provide an adequate supply [1]. Se deficiency has been reported in regions of the world with selenium-poor soils used for agriculture, commonly in parts of Russia, China, and Europe. The increased incidence of gastrointestinal pathologies, including inflammatory bowel disease, chronic diarrhea, bowel resections for colon cancer, bariatric surgery (for weight loss), and long-term use of parenteral nutrition deficient in Se has led to an increased number of reported cases of Se deficiency in the USA [2-5]. Dilated cardiomyopathy secondary to Se deficiency (i.e., Keshan disease) is a well-recognized outcome and occasionally reversed by Se supplementation [6]. Se primarily exerts its effects via incorporation into selenoproteins, a wide variety of selenium-dependent enzymes with roles ranging from an antioxidizing agent, regulating the inflammatory responses, and causing the proliferation and differentiation of immune cells [7]. We report the case of an unfortunate woman with severe malnutrition due to bowel resection from inflammatory bowel disease who presented with dilated cardiomyopathy and reduced ejection fraction (EF), which improved on parenteral Se administration.

## Case presentation

A 53-year-old woman was admitted to our hospital with a three-day history of epigastric pain, nausea, vomiting, and increased ileostomy output. She denied fever, chills, cough, shortness of air, hematemesis, hematochezia, melena, or urinary symptoms. Her past medical history was significant for metastatic melanoma, Crohn’s disease, severe malnutrition, hypothyroidism, and congenital ichthyosis. Past surgical history included melanoma excision, cholecystectomy, partial hepatectomy for metastatic melanoma, hysterectomy, oophorectomy, total colectomy with end ileostomy, and Port-A-Cath placement. She denied cigarette smoking or illicit drug use but reported occasional alcohol use. Physical examination revealed a cachectic woman with a body mass index of 15 kg/m^2^ in no acute distress. She had a heart rate of 117, and other vital signs were normal. She had decreased air entry on lung bases, no heart murmur or peripheral edema, a soft, but distended abdomen with hypoactive bowel sounds, and an ileostomy in place that appeared intact. Other systems examined were otherwise unremarkable. Her troponin levels were normal. Other laboratory data showed leukocytosis with a left shift but no bandemia, normal lactic acid levels, elevated blood urea nitrogen and creatinine, and low prealbumin. Her serum Se levels were markedly reduced (65 ug/L). Her electrocardiogram (EKG) showed sinus tachycardia with anterolateral ST depression and T wave inversion. A transthoracic echocardiogram (TTE) was done to evaluate these EKG findings and revealed left ventricular systolic dysfunction with an EF of 25% and global hypokinesia. An abdominal computed tomography (CT) scan showed dilated loops of bowel with no clear transition point, and an esophago-gastro-duodenoscopy revealed a duodenal ulcer and mild diffuse esophagitis. The patient was allowed nothing by mouth and started on intravenous (IV) fluids for presumed ileus, empiric ciprofloxacin, and metronidazole for possible urinary versus gastrointestinal tract infection. She was given fluconazole for likely candida esophagitis and a proton pump inhibitor for her duodenal ulcer. Since abdominal symptoms resolved and cultures did not yield any growth, antibiotics were later discontinued, and total parenteral nutrition was started for severe malnutrition. She was discharged on a wearable defibrillator and a three-week course of IV Se at 100 ug/day. A beta-blocker and angiotensin-converting-enzyme inhibitor were not started due to her low blood pressure (90/60 mmHg). A one-month follow-up TTE revealed an EF of 40%.

## Discussion

The dietary reference intake for Se recommends a dose of 55 ug/day for adults. Patients on total parenteral nutrition (TPN) who are Se-deficient may require up to 150 ug/day of supplemental Se [8]. Given Se’s widespread availability over the counter in the US, a deficiency of this essential element is therefore due to malnutrition occasioned by malabsorption due to chronic diarrhea, inflammatory bowel disease, bowel resection for malignancy, bariatric surgery, and patients on Se-deficient TPN. Se is incorporated in selenoproteins, and over 30 different kinds of selenoproteins have been described in the literature [7]. In the cardiovascular system, glutathione peroxidases have a clear role in the regulation of oxidative stress. Cytosolic glutathione peroxidase-1 has shown to have an important role in detoxifying intracellular reactive oxygen species and protects against oxidative damage from the ischemia-reperfusion cycle. When incorporated into the various selenoenzymes, the increased antioxidant capacity suppresses the production of interleukins and tumor necrosis factor alpha in cardiomyocytes. Therefore, its deficiency leads to endothelial dysfunction with consequent structural abnormalities in vascular and cardiac tissues [9]. Heart failure induced by Se deficiency is well-described in the literature with the earliest cases being described in 1935 [6]. Keshan disease, a dilated cardiomyopathy affecting primarily children and nursing mothers, was first described in parts of China, where the soil’s deficiency in Se lead to a diet deficient in Se. A randomized controlled study with Se supplementation reported a reduction in morbidity and mortality [6]. To et al. described three cases of severe dilated cardiomyopathy in intubated patients receiving parenteral nutrition that improved after Se supplementation [4]. Massoure et al. described the case of a 40-year-old patient presenting with dilated cardiomyopathy and Se deficiency following bariatric surgery with a resolution of symptoms after Se repletion [2]. Saliba et al. described a case of Se-deficient cardiomyopathy that was reversible after supplementation [10]. Unfortunately, our patient had a combination of Crohn’s disease, colectomy, and TPN, all of which led to Se deficiency. Her symptoms and EF improved after supplementation despite not being started on heart failure medications.

## Conclusions

Patients with malnutrition and signs of heart failure should be screened for a deficiency in micronutrients such as Se, as cardiomyopathy may be reversible with Se supplementation.
